# Transcriptomic landscape of blood platelets in healthy donors

**DOI:** 10.1038/s41598-021-94003-z

**Published:** 2021-08-03

**Authors:** Anna Supernat, Marta Popęda, Krzysztof Pastuszak, Myron G. Best, Peter Grešner, Sjors In ’t Veld, Bartłomiej Siek, Natalia Bednarz-Knoll, Matthew T. Rondina, Tomasz Stokowy, Thomas Wurdinger, Jacek Jassem, Anna J. Żaczek

**Affiliations:** 1 Laboratory of Translational Oncology, Intercollegiate Faculty of Biotechnology, University of Gdańsk and Medical University of Gdańsk, Dębinki 1, 80-211 Gdańsk, Poland; 2 Department of Algorithms and Systems Modelling, Faculty of Electronics, Telecommunications and Informatics, Gdańsk University of Technology, Gdańsk, Poland; 3 Department of Neurosurgery, Brain Tumor Center Amsterdam, Cancer Center Amsterdam, Amsterdam UMC, VU University Medical Center, Amsterdam, The Netherlands; 4 Brain Tumor Center Amsterdam, Amsterdam UMC, VU University Medical Center, Amsterdam, The Netherlands; 5 Department of History and Philosophy of Medical Sciences, Medical University of Gdańsk, Gdańsk, Poland; 6 University of Utah Molecular Medicine Program, Salt Lake City, UT USA; 7 Department of Internal Medicine, Division of General Internal Medicine, University of Utah, Salt Lake City, UT USA; 8 George E. Wahlen Veterans Affairs Medical Center Department of Internal Medicine and the Geriatric Research Education and Clinical Center (GRECC), Salt Lake City, UT USA; 9 Department of Pathology, University of Utah, Salt Lake City, UT USA; 10 Department of Clinical Science, University of Bergen, Bergen, Norway; 11 Department of Oncology and Radiotherapy, Medical University of Gdańsk, Gdańsk, Poland

**Keywords:** Molecular biology, Physiology, Systems biology, Medical research, Molecular medicine, Mathematics and computing

## Abstract

Blood platelet RNA-sequencing is increasingly used among the scientific community. Aberrant platelet transcriptome is common in cancer or cardiovascular disease, but reference data on platelet RNA content in healthy individuals are scarce and merit complex investigation. We sought to explore the dynamics of platelet transcriptome. Datasets from 204 healthy donors were used for the analysis of splice variants, particularly with regard to age, sex, blood storage time, unit of collection or library size. Genes *B2M, PPBP, TMSB4X, ACTB, FTL, CLU, PF4, F13A1, GNAS, SPARC, PTMA, TAGLN2, OAZ1* and *OST4* demonstrated the highest expression in the analysed cohort, remaining substantial transcription consistency. *CSF3R* gene was found upregulated in males (fold change 2.10, FDR q < 0.05). Cohort dichotomisation according to the median age, showed upregulated *KSR1* in the older donors (fold change 2.11, FDR q < 0.05). Unsupervised hierarchical clustering revealed two clusters which were irrespective of age, sex, storage time, collecting unit or library size. However, when donors are analysed globally (as vectors), sex, storage time, library size, the unit of blood collection as well as age impose a certain degree of between- and/or within-group variability. Healthy donor platelet transcriptome retains general consistency, with very few splice variants deviating from the landscape. Although multidimensional analysis reveals statistically significant variability between and within the analysed groups, biologically, these changes are minor and irrelevant while considering disease classification. Our work provides a reference for studies working both on healthy platelets and pathological conditions affecting platelet transcriptome.

## Introduction

Circulating blood platelets are anucleate cytoplasm fragments, derived from megakaryocytes. Once released, they circulate in the bloodstream for up to 10 days, playing a crucial role in the maintenance of homeostasis and regulation of inflammation^1,2^. Platelets are composed of a cell membrane, organelles, elements of cytoskeleton and granules. Equipped with mitochondria but devoid of the nucleus, they lack genomic DNA, remaining incapable of de novo transcription^3–5^. Thus, they rely on a rich repertoire of RNAs, complemented by the presence of translational machinery, which allows translation of nascent transcripts into proteins under environmental stress^6^. Platelets host a broad spectrum of transcripts, including a variety of messenger RNAs, diverse classes of non-coding RNAs, microRNAs and circular RNAs^3,5^.

Historically, first studies on platelet RNA content involved Northern blot hybridization method, later replaced with a more robust RT-qPCR (Reverse Transcription quantitative Polymerase Chain Reaction). Along with the implementation of microarrays, it became feasible to further characterize the platelet transcriptome^7,8^. The introduction of RNA sequencing (RNA-seq) provided even more detailed information on the number and biotypes of RNAs in platelets. This method, combined with platelet sorting, allowed investigators to characterize and compare young, newly released platelets to platelets that had been circulating for a longer period of time^9^. The ultimate golden trove for platelet transcriptome analysis remains single platelet sequencing. This approach, however, is challenging due to the limited amount of input material, with one platelet estimated to have ~ 2.2 femtograms of total RNA mass^10^. Currently, only single megakaryocyte RNA sequencing is feasible^11,12^.

While maintaining homeostasis and mediating inflammation, platelets are implicated in other pathophysiological processes. Their function, and thus content, might be affected by the ongoing disease, and the list of ailments with altered platelet transcriptome keeps expanding. A large number of studies concerns heart conditions and cancer. Aberrant RNA profiles have been discovered in cardiovascular disease^13^, atrial fibrillation^14^, acute myocardial infarction^15^, as well as in myeloproliferative neoplasms^16^, and solid tumours^17,18^. In the latter case, platelets have even earned a separate name: TEPs (Tumor Educated Platelets). An altered platelet transcriptomic profile has also been observed in sickle cell disease^19^ and sepsis^20^.

In healthy humans, the number of blood cells remains tightly controlled within narrow physiologic ranges, with cytokine-mediated signalling and transcriptional circuits regulating hematopoietic dynamics^21^. The platelet transcriptome is considered stable in healthy individuals over time^22^. Nevertheless, RNA expression in hematopoietic stem cells is subject to changes as the cells age^23^ or become exposed to sex hormones^24^. Thus, we wondered about the extent of dynamics in the platelet transcriptional profile. Available reports concerning this subject included a limited number of samples or used healthy donor samples as a frame of reference, not as the direct subject of study^25–28^. As platelet RNA-seq is increasingly used by groups around the world, we sought to combine and analyze platelet RNA-sequencing data sets from 204 healthy donors. We hypothesized that the highest ever reported number of samples would enable us to more robustly identify and control for variability in the platelet transcriptome which might not otherwise be apparent in smaller datasets. We analyzed platelet transcriptome with respect to the age, gender or technical variables, such as sequencing library size, storage time, and blood collection conditions (collecting unit). In addition to investigating separate RNA splice variants, we also included multidimensional sample vector analysis. Our work might be considered a reference for studies on both healthy platelets and different pathological conditions.

## Materials and methods

### Study cohort

Hoping to refine the current knowledge on the platelet transcriptome landscape, we included samples collected from 204 asymptomatic controls. These were all the samples available at the moment of analysis. Assuming the variance of splice variant expression levels in platelets of approximately 15% (interquartile range: IQR of 11–19%), statistical power of 80%, the level of statistical significance of 0.05 and the use of double-sided statistical tests, such sample size was far enough to indicate between-group statistically significant differences as little as only 5% (IQR 4–7%). It is important to emphasize that when comparing biological differences among the studied subgroups, we were focusing on the expression changes of at least 50%, while considering at least twofold (100%) differences as possibly biologically meaningful.

**Figure 1:**
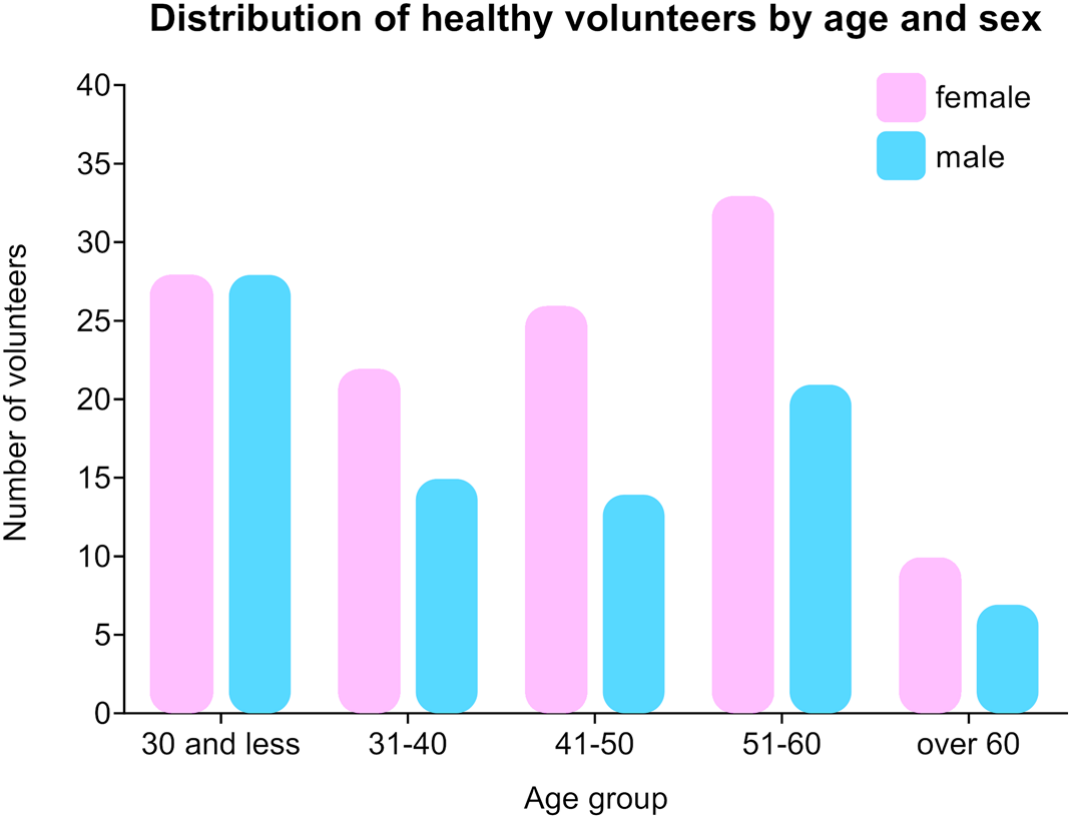
Age and sex distribution of healthy donors.

All the donors provided written informed consent and reported to be disease-free. The individuals were not subjected to extensive diagnostic tests to rule-out undiagnosed conditions. Whole blood was collected into 6-mL EDTA Vacutainer tubes and then processed according to thromboSeq protocol published before^29^, with the majority of the samples processed between 2 and 12 h after collection. The study was conducted in accordance with the principles of the Declaration of Helsinki. Approval for this study was issued by the Medical Ethical Committee, VU University Medical Center, Amsterdam, The Netherlands. The detailed list of cases is presented as Supplemental Table S1. RNA-seq data is available at Gene Expression Omnibus (GEO) under accession number: GSE89843. Prior to bioinformatics processing, all samples passed bioinformatics quality checks included in thromboSeq protocol^29^. In brief, the intron-spanning spliced RNA reads were selected to eliminate possible DNA contamination. Next, gene body coverage analysis was performed. We additionally applied a cut-off of 100 k reads per sample to improve the reliability of results. Our cohort consisted of 195 samples collected at the Amsterdam UMC VU University Medical Center and 9 samples collected in Amsterdam UMC Academical Medical Center Amsterdam. One hundred nineteen patients were female, and 85 were male. Details on age and sex distribution in the cohort are presented in Fig. 1. The reason why more females than males were included in the dataset is due to the type of study this reference cohort was collected for. All donors were adult, representing all age groups (range 18–86). The mean age was 42.11 years [standard deviation (SD) 13.890], and the median age was 42.5 years (IQR 29–53). Samples were sequenced in several, randomly allocated batches between November 2015 and July 2016.

### Processing of RNA-seq data

Raw RNA-seq data encoded in FASTQ-files were subjected to a standardized RNA-seq alignment pipeline, as described previously^17,29^. Briefly, reads were subjected to trimming of sequence adapters by Trimmomatic (v. 0.22), mapped to the human reference genome (hg19) using STAR (v. 2.3.0), and summarized using HTSeq (v. 0.6.1), guided by the Ensembl gene annotation version 75. Samples with less than 100 k total reads were excluded from further analysis. Ensemble IDs were converted into gene names using Gencode v19 GRCh37 annotation. Genes with known status were accepted for further analysis. In cases with two IDs mapped to the same gene name, level 1 records were used (Comprehensive gene annotation files, Gencode).

### Statistical analysis

Subsequent analysis of generated read counts was performed in R (version 3.6.1) and R-studio (version 1.2.5019). For normalization, the DESeq2 R package^30^ with Variance Stabilizing Transformation^31^ was used. Transcripts with mean log2 count below four in all groups were excluded. Basic statistical analysis involved t-tests with Benjamini & Hochberg false discovery rate (FDR) correction^32^. Mean-based fold changes (FC) between age-sex-related groups and sample clusters were evaluated. Correlation between spliced junction reads and age was estimated with the Pearson correlation coefficient. Unsupervised clustering of the samples was performed using Ward D2 agglomerative clustering algorithm^33^ as implemented in R package stats (source: https://www.rdocumentation.org/packages/stats/). Heatmaps were generated for the most differing transcripts using heatmap3 R package (source: https://cran.r-project.org/web/packages/heatmap3/). Selected transcripts were functionally annotated with Reactome pathways using over-representation analysis tool by ConsensusPathDB^34^. Interactions between protein products of selected transcripts were visualized using STRING v11^35^. Canonical platelet transcripts were defined according to the platelet markers listed in PanglaoDB^36^. To further assess variability, splice variants were analysed globally as multidimensional vectors. Variability in platelet transcriptome was assessed by testing the between-group differences in respective mean RNA transcript vectors, between-group differences in within-group multidimensional RNA transcript vector distribution, and between-group differences in within-group multidimensional similarity among RNA transcript vectors. The between-group differences in mean splice variant vectors and their within-group multidimensional distribution were analysed using the gene set analysis approach by means of the two-sample nonparametric multivariate test of means based on the minimum spanning tree and Kolmogorov–Smirnov statistic and the multivariate generalization of the Wald–Wolfowitz runs test, respectively, as implemented in the GSAR R package obtained from Bioconductor. Within-group multidimensional similarity among splice variant vectors was calculated as median reciprocal to all pair-wise Euclidean distances among all possible pairs of RNA splice variant vectors from a given group. Statistical significance of between-group differences in such multidimensional within-group similarity among RNA transcript vectors was assessed by testing the difference between median similarities in the compared groups against its permutational distribution obtained from 10,000 permutations. Statistical significance was inferred for p < 0.05, with Bonferroni correction for multiple testing where necessary.

## Results

### General characterization of platelet transcriptome in healthy individuals

Expression of spliced junction reads in human platelets ranged up to six orders of magnitude. The average number of reads per sample was 811,857.6 in the evaluated cohort of 204 healthy donors. Initially, 57,736 different splice variants were mapped. As the quality of the mapped transcripts varied, and a number of them were expressed only in a small subset of samples, reads were subjected to quality control and filtering of low expression RNAs, using the exclusion criteria of normalized log2 counts below four in all groups. Eventually, 3954 transcripts were qualified as appropriate for further analysis. RNA biotypes before and after filtering are presented as Supplementary Fig. S1. Importantly, SMARTer Kit used in the sequencing protocol enriches for poly-A tailed RNAs, which affects the types of RNAs identified.

**Figure 2:**
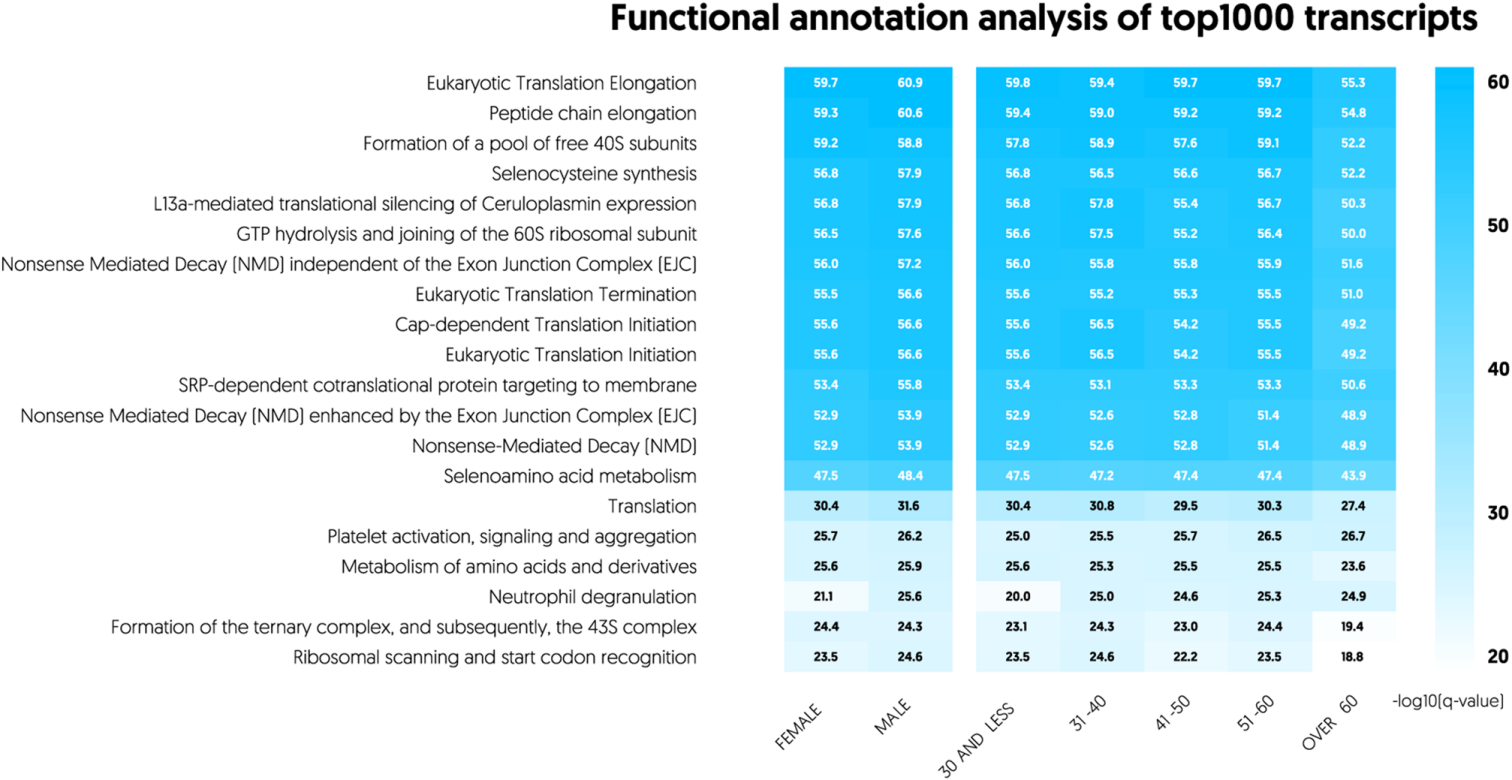
Functional annotation analysis of top expressed spliced genes among the analysed sex and age groups. For each group, 1000 spliced genes with the highest expression level were subjected to over-representation analysis and top20 Reactome pathways were identified based on − log10(q-value).

Most of the analysed variants in the cohort included mRNAs (98.6%), with a small number of pseudogene transcripts (1.1%). Genes *B2M*, *PPBP*, *TMSB4X*, *ACTB*, *FTL*, *CLU*, *PF4*, *F13A1*, *GNAS*, *SPARC*, *PTMA*, *TAGLN2*, *OAZ1* and *OST4* demonstrated the highest expression in the dataset, retaining substantial expression stability (mean log2 normalized counts > 12, SD range 0.51–1.04, Supplementary Table S2). STRING analysis revealed a strong network interaction occurring between the protein products of these 14 transcripts, as shown in Supplementary Fig. S2. These mRNAs were heavily involved in vesicle-mediated transport and secretion. Further functional annotation analysis of enriched Reactome pathways (CPDB, ConsensusPathDB database) demonstrated that the top 1000 transcripts were typically involved in protein synthesis and platelet activation signalling, as presented in Fig. 2. For clarity of depiction, we are showing only the first top 20 expressed pathways. We also examined the variance in the expression of spliced junction reads in our cohort. Not only were the platelets of healthy donors consistent in terms of canonical platelet splice variants defined according to PanglaoDB (SD range 0.44–1.94), but also remained considerably stable across the remaining non-canonical transcripts (SD range 0.29–2.57) (Supplementary Table S2).

### Platelet splice variants according to sex and age

When comparing transcriptomic profiles between males and females, mean spliced reads expression analysis per chromosome confirmed that the major discrepancy was observed for chromosome Y (Supplementary Fig. S3). Except for Y-specific differences, the female and male platelet transcriptome demonstrated substantial similarity, reflected by 94.4% overlap of top 1000 transcripts in both groups. Only one gene—*CSF3R*, coding for Colony Stimulating Factor 3 Receptor, was found significantly upregulated in males when compared to females (FC = 2.10, FDR-corrected q-value < 0.05). We also observed a moderate upregulation (FC > 1.5, FDR-corrected q-value < 0.05) of another 82/3954 (2.1%) transcripts that were predicted to be involved in neutrophil-mediated immune response and extracellular matrix organization (Supplementary Table S3).

**Figure 3:**
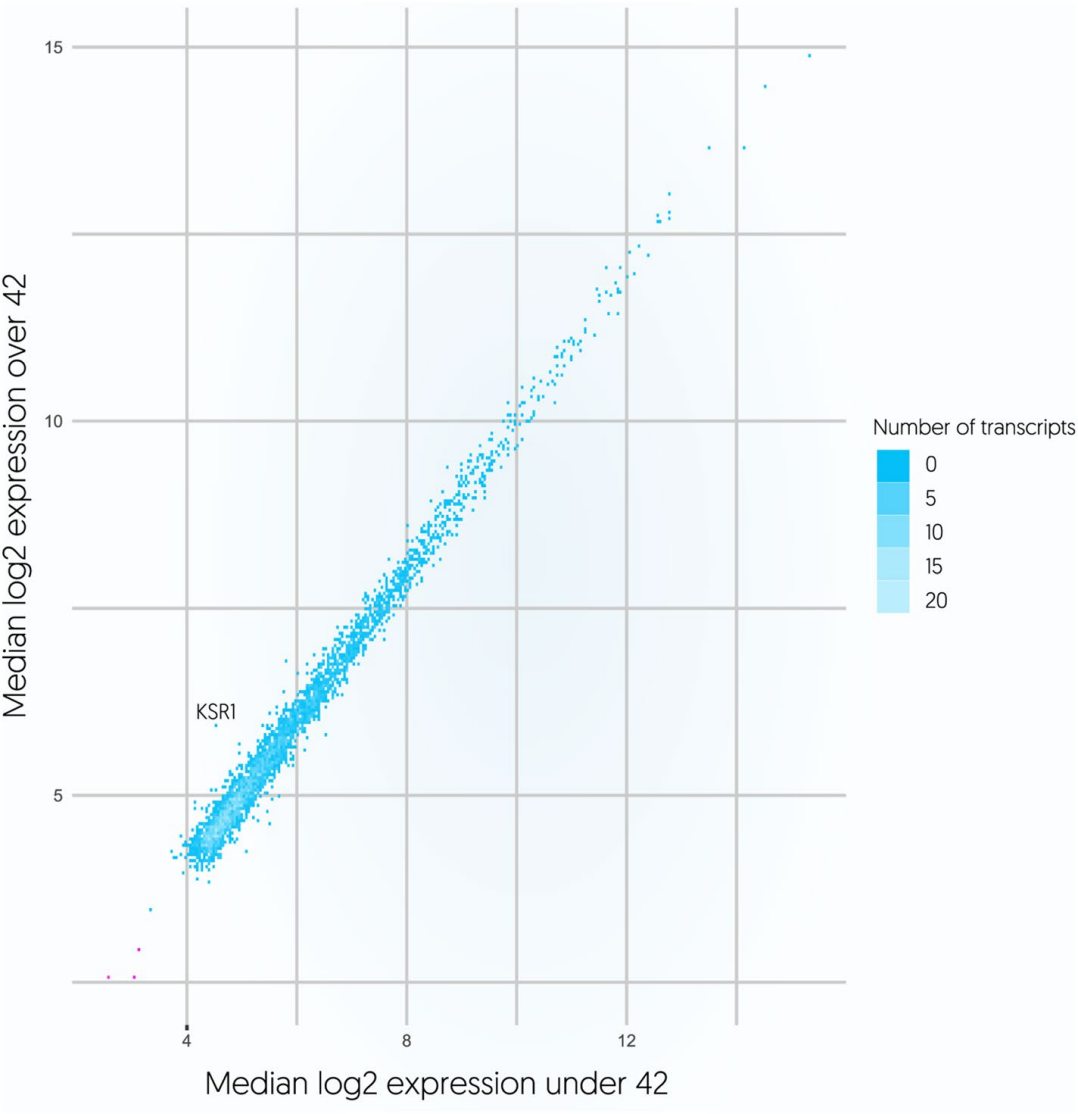
Plot representing the relation between log2 median spliced platelet RNA profile of donors younger (X axis) and older than 42 years (Y axis).

**Figure 4:**
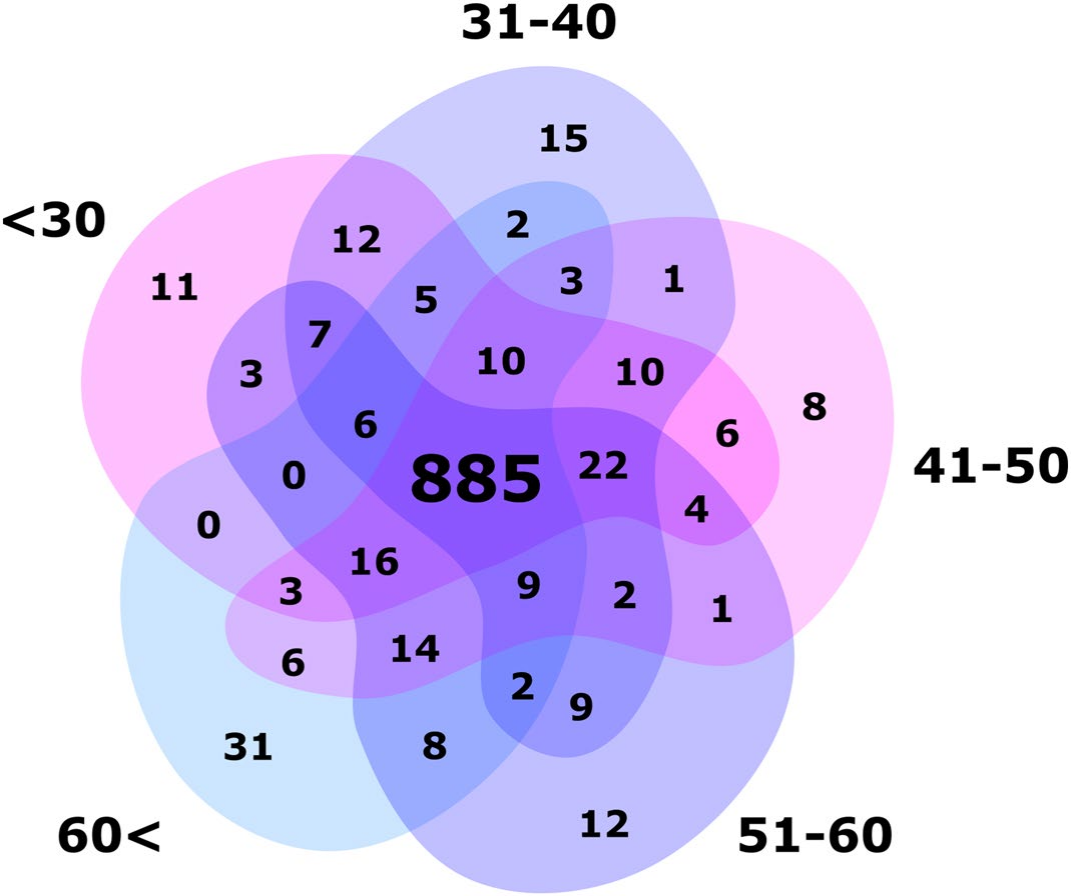
Top 1000 splice variants notably overlap between the studied five age groups.

We next investigated the effect of ageing on the platelet transcriptome. Direct correlation analysis demonstrated a modest association (r > 0.3 and r < − 0.3) between the expression of 32/3954 (0.8%) transcripts and age. The gene list included: *KSR1, STXBP2, CAPZB, KIAA2013, FCGR2A, PPIF, TALDO1, MGAT4B, RAB6B, SF3A2, GPSM3, CAPNS1, ZYX, TMED3, TMSB4X, RAB11A, BROX, UBE2K, CALM1, TADA2A, THOC2, TRAPPC2, OPA1, HIST1H4H, EIF5, CALM2, AP1S2, HIST1H2BC, STK4, MAP1LC3B, CARD8* and *KRAS*. The highest coefficient in the whole dataset was observed for the gene *KSR1* (r = 0.409, FDR corrected q value < 0.001) encoding Kinase suppressor of Ras 1. Cohort dichotomization, according to the median age (42.5 years) has confirmed that *KSR1* was the only significantly differing gene between the older and younger participants (FC = 2.11, Fig. 3). When considering 50% differences in spliced read counts, 4 transcripts—*HDAC8, TGFB1, FOS* and *SAMD14*, were upregulated (FC > 1.5) and 4 transcripts—*IL7R, LEF1, HIST1H4H* and *ANXA3*, were downregulated (FC < 0.67) in older versus younger donors (all FDR corrected q value < 0.05). Nonetheless, analysis of mean spliced reads per chromosome revealed substantial consistency among younger and older donors. To further explore the dynamics of transcriptome variability in donors we applied various age cut offs (Supplementary Figs. S4–7). This additional discrimination confirmed considerable homogeneity across splice variants analysed separately, with high top 1000 transcript overlap (88.5%) between 5 age subgroups (Fig. 4).

### Global variability of platelet transcriptome

**Figure 5:**
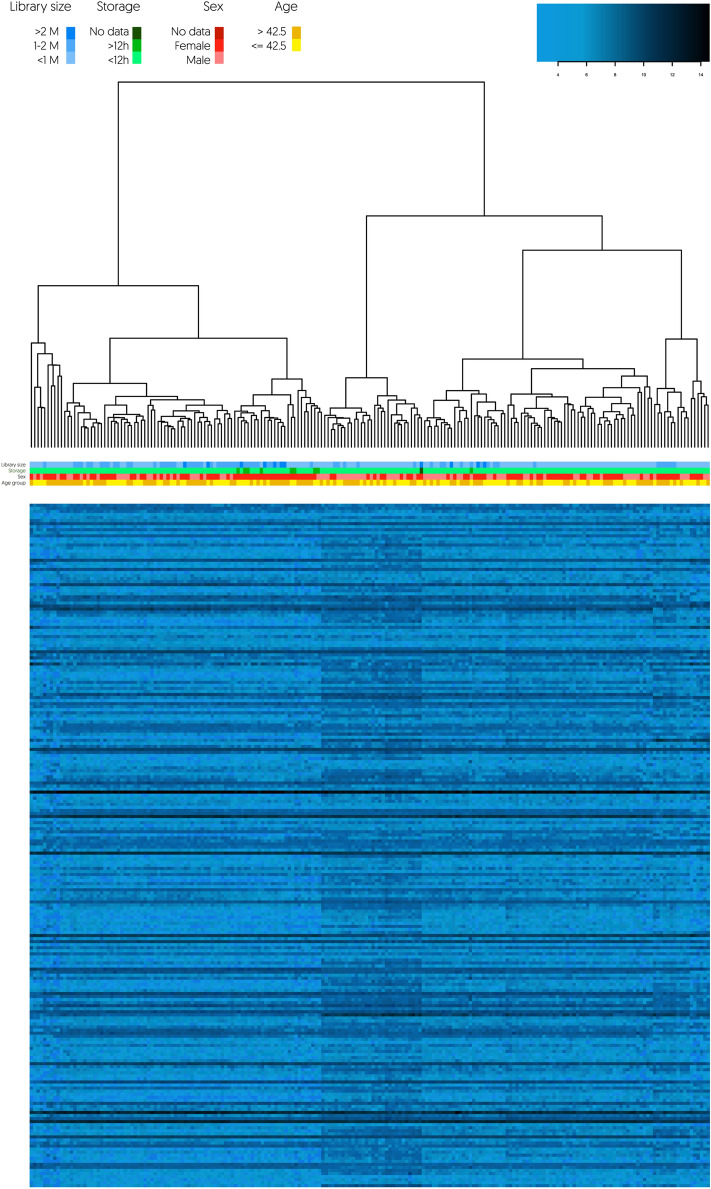
Heatmap depicts the expression of 224 spliced genes with the greatest, at least fourfold mean changes between the two clusters revealed by unsupervised hierarchical clustering. Colour legend indicates the sex, age group, blood collection unit, storage time and library size of each sample. The clusters presented substantial similarity in terms of the expression of spliced junction reads. 62% of spliced platelet RNAs demonstrated less than a twofold expression difference. Only 16% of spliced genes in the dataset (649/3954) demonstrated at least a threefold expression difference, while at least fourfold difference was observed in only 6% of the spliced reads (224/3954). Due to prior clustering, all these observations were statistically significant (FDR corrected q value < 0.05).

To further explore platelet transcriptome, we decided to verify whether there was any other variability within the analysed dataset. Unsupervised hierarchical clustering revealed two clusters of samples, depicted in Fig. 5. In this process of unsupervised clustering, samples are assigned to groups with respect to their expression profile, not parameters such as sex or age. Eventually, samples similar to one another in terms of splice variant expression are clustered together, while samples with differing expression profiles will be clustered apart. This means that clusters are built based on the similarity of subsets of samples. Importantly, all the analysed factors (sex, age, unit of blood collection or library size) were randomly distributed across both clusters. Hence, there was no bias towards any of the clusters with respect to sex, age, unit of blood collection or library size. This suggests that these variables did not affect platelet transcriptome considerably. Of note, all the samples passed our QC filtering criteria (the poorest quality analysed was 130,709 reads). Intriguingly, all of the differentially expressed spliced junction reads with at least fourfold difference were upregulated in cluster 2. According to functional analysis, those 224 genes were involved in processes related to neutrophil-mediated immune reaction and interactions at the vascular wall, as depicted in Supplementary Fig. S8. To this end, we named the first cluster “Immune cold”, while the second “Immune hot”. There were 6 splice variants (*FCN1*, *ITGB2*, *LYZ*, *PTPRC*, *CTSS* and *CPVL*) with over tenfold difference between the clusters, all associated with neutrophil degranulation process.

Noteworthy, the clusters demonstrated a substantial similarity in terms of the most abundant transcripts. At this stage, we inspected the platelet RNA profiles manually, by looking at the top 100 spliced genes in each cluster. Altogether, we identified 134 unique splice variants, with an overlap of 78 transcripts and 28 transcripts specific for each cluster. The top 78 spliced genes common for both clusters were mainly platelet-specific, involved in haemostasis, cell–extracellular matrix interactions and smooth muscle contractions processes (CPDB). When focusing on differences, top spliced reads unique for cluster “Immune cold” were implicated in the synthesis and metabolism of fatty acids, whereas those unique for cluster “Immune hot” were associated with translation processes.

Next, we assessed the variability of platelet transcriptome considering each donor’s transcriptome a multidimensional vector and analysed between and pithing group variability (Table 1). Statistically significant differences in multidimensional mean RNA transcript vector were found between groups obtained based on gender, storage time, unsupervised Ward-based hierarchical clustering, and library size. The within-group multidimensional distribution of RNA transcript vectors was found to differ between groups obtained based on gender, age, clustering, median, and processing time. The within-group similarity among RNA transcript vectors were found to differ between groups obtained based on clustering, library size, and processing time. Detailed numerical data are available as Supplementary Table S4.

**Table 1:** Summary of analysis of variability associated with selected factors. Values below the assumed thresholds are marked in bold.

Analysed parameter potentially affecting platelet transcriptome	Mean splice reads vectors (p_KS_)	Within-group splice read distribution (p_WW_)	Within-group similarity among splice read vectors (p)
**Gender**			
Female vs. male	**0.0186**^**a**^	**0.0010**^**a**^	0.8502
**Age (Group division I)**			
< 30 vs. 31–40	0.7012	0.3497	0.0196
< 30 vs.41–50	0.3761	0.1009	0.7144
< 30 vs. 51–60	0.3843	0.2348	0.0552
< 30 vs. > 60	0.8304	0.0140	0.9676
31–40 vs. 41–50	0.6157	0.0809	0.3815
31–40 vs. 51–60	0.9509	0.1838	0.0259
31–40 vs. > 60	0.6137	0.1968	0.7439
41–50 vs. 51–60	0.0653	0.5475	0.3360
41–50 vs. > 60	0.4794	0.5894	0.0234
51–60 vs. > 60	0.7843	0.9341	0.7600
**Age (Group division II)**			
< 40 vs.41–60	0.6985	**0.0130**^**a**^	0.2115
< 40 vs. > 60	0.8724	0.1189	0.2544
41–60 vs. > 60	0.3450	0.9151	0.0240
**Age (median)**			
< 42 vs. > 42	0.5674	**0.0020**^**a**^	0.1150
**Storage time**			
< 12 h vs. > 12 h	**0.0010**^**a**^	0.0629	0.0631
**Cluster**			
Cluster 1 vs. Cluster 2	**0.0010**^**a**^	**0.0010**^**a**^	**0.0004**^**a**^
**Library size**			
1 vs. 2	**0.0005**^**b**^	0.2398	**<< 0.0001**^**b**^
1 vs. 3	**0.0029**^**b**^	0.5225	**0.0013**^**b**^
2 vs. 3	**0.0159**^**b**^	0.6943	0.5301

^a^p_threshold_ = 0.0500.

^b^p_threshold_ = 0.0167.

## Discussion

As great efforts are invested in translating the sequencing of liquid biopsies into clinical practice, it is becoming imperative to provide detailed information on RNA blood content. The available literature on healthy platelet transcriptome mostly relies on a limited sample size^22,25,27,28^. Small sample size is bound to be prone to high FDR q values, and thus conclusions need to be derived with caution. We did not compare our results directly to other publicly available datasets^22,37^ as these data differed from our study with respect to the: participant enrolment procedure, blood processing protocol, RNA extraction protocol, RNA reverse transcription, library preparation, sequencing settings, bioinformatic pipeline and eventually, splice variant filtering criteria. In the presented study, which included over 200 samples, we have shown consistency of separately analysed splice reads which rarely surpassed criteria of statistical significance. Homogeneity regarding fold changes prevailed in direct comparison (female versus male, young versus old), with a few consistent tendencies (increase or decrease) across the analysed sex and age groups: *CSF3R*, *KSR1*, *IL7R*, *TGFB1*, *IGTA2B*. Despite these tendencies, a considerable expression overlap would be observed between the studied groups. Interestingly, Segal and Moliterno reported platelet counts to vary according to age, sex, and also ethnicity^38^. Their study performed on 12,142 donors has shown a very small but steady decline in platelet counts with age, the higher platelet count in women, and the lowest platelet count in whites when compared to other ethnicities. We found rather minute differences among age and sex groups, with considerable overlap in splice variant expression between the studied sub-cohorts.

Age-related changes in platelet transcriptome were published before^39,40^. Campbell et al. identified 514 differentially expressed transcripts, with most (455) being increased in older adults aged 65 years and older. However, the exact same transcripts, when analysed in a large cohort of our study, would range only between 0.61 to 1.46 in terms of fold change (when over 60 and 40 and less groups were compared). Importantly, as the study of Campbell et al. validated the expression and function of Granzyme A by multiple transcriptomic, proteomic, and functional biochemical assays, the discrepant findings are likely the consequence of different sequencing techniques, analytics, filtering methods, and healthy donor populations.

Another interesting age-related study included six adults and six neonates, with 201 genes found differentially expressed^41^. According to this report, neonatal platelets would contain high RNA levels of transcripts associated with protein synthesis and processing, simultaneously carrying lower levels of genes involved in calcium transport, calcium metabolism and cell signalling. Our study did not contain subjects younger than 18, but then again, the low sample size could be the source of bias in the aforementioned report.

The study of Edelstein et al. reported racial differences in human platelet transciptome, with a very thoroughly characterised healthy volunteer group of 154 donors^26^. Unfortunately, our data lacked information on ethnicity. However, the included samples were collected in Amsterdam. Hence, roughly half of the donors were likely to be Dutch, with most of the remaining participants of European, Maroccan, Surinamese and Turkish descent. Additionally, our study was based on RNA-seq, not microarrays. As much as it renders any comparisons challenging, it is worth noting that, contrary to our observations, Edelstein et al. reported multiple mRNAs and miRNAs to be differentially expressed between the compared groups. These discrepancies are likely to stem from our assumed strict filtering criteria, adjusted to thromboSeq classification^29^. Any compromise in filtering would yield lower quality and thus the credibility of the performed analysis. Of note, the presented QC eliminated most of the protein-coding transcripts, which were reported to have low expression, and all miRNAs sequenced.

Our unsupervised hierarchical clustering revealed two, relatively evenly distributed clusters of samples. Importantly, the clusters were irrelevant of age, sex, or the unit of blood collection. We hypothesise that patients belonging to the “Immune hot” cluster may have been undergoing covert infection. Apart from all this stated above, the landscape of platelet transcriptome presents slightly differently when donors’ transcriptomes are analysed globally, as multidimensional vectors. To the best of our knowledge, there are no previous studies reporting expression data as vectors. In such analyses, factors such as gender, storage time, library size, the unit of blood collection as well as age and processing time were identified as factors capable of introducing between-group variability in the mean distribution of RNA splice variant vectors and/or the within-group similarity among them. Importantly, while statistically significant differences need to be acknowledged, biological significance of these changes seems of far lesser importance. Hence, the transcriptome of healthy subjects is exquisitely durable while being dynamically altered in the state of disease, as also highlighted by Davizon-Castillo et al.^11^. Consequently, artificial intelligence-based classifiers such as thromboSeq or imPlatelet, while performing discrimination between healthy and diseased individuals appear irrelevant of variables such as age or sex^18,42^.

One of the limitations of the study was the lacking information on platelet count of each donor. It might be speculated that re-scaling of the obtained read counts accounting for this variable, or factors such as menopause status or hormone replacement therapy, would refine the performed analysis, demonstrating even further consistency. Unfortunately, sample enrolment could not rule out cases with undiagnosed cancer, an ongoing inflammatory or infectious disease. Due to the anonymization of the sample collection procedure, we could not trace back the healthy donors who donated blood for clinical follow-up. Another limitation was the application of RNA-seq approach only, without further validation by other methods, such as RT-qPCR or proteomics analysis. It is also crucial to point out the weaknesses of the applied method: used reagents enrich for polyA splice variants, whereas bioinformatic pipeline causes the loss of one-exon transcripts and miRNAs which might also be of relevance in the disease process. The last important limitation was an occasional long period between blood collection and processing. However, we did not observe any crucial variability among the samples which were processed later than 12 h after blood collection. This information is important as high stability makes platelets collected in EDTA-coated tubes a very convenient liquid biopsy material. Finally, an important feature for platelet transcriptome analysis is the risk of leukocyte contamination. It has been previously shown that contamination of such nucleated cells using platelet isolation protocol is low^17,18,29^—also without significant platelet activation—though we cannot rule out co-isolation of other cell fragments of similar size as platelets.

In summary, platelets’ transcriptome carries diagnostic information relevant in conditions such as cancer or cardiovascular disease. As reference data on the platelet transcriptome in healthy individuals are still scarce and merit complex investigation, we performed an in-depth analysis of platelet transcriptome landscape. Our study, based on a large dataset, proves that healthy human platelets have a unique and reproducible transcript profile. The knowledge on the transcriptome landscape can be used for example in RT-qPCR setting, where top spliced genes, such as *B2B*, *PPBP* and *ACTB*, could potentially constitute reference genes when analysing gene expression. Platelet profile demonstrates consistency, which remains an important implication when recruiting a control cohort for any experiments. We show that separately analysed platelet splice variants retain general consistency, with a few interesting deviating genes from these general trends. When assessed globally (as vectors representing corresponding samples), factors as sex, age, storage time or library size impose a certain degree of between- and/or within-group variability. However, for disease classification purposes, especially cancer, these differences remain negligible^18,42^. The presented results suggest that, when planning an oncology-related study, control cases do not necessarily need to be sex or age-matched. Ideally, they should be, but even without the matching, the classification most probably will not be affected to a classification output extent. Similarly, blood samples should be processed as soon as possible after collection, in room temperature (to avoid platelet activation), but even when processed after over 12 h, the results are still meaningful. This is especially important when considering logistic aspects of diagnostics. In many cases blood material is not directly processed at the hospital, but rather transported prior to analysis. To conclude, factors such as age or storage time introduce small variability, unlikely to have strong biological effects.

## Supplementary Information

Supplementary Figures.

Supplementary Table S1.

Supplementary Table S2.

Supplementary Table S3.

Supplementary Table S4.
