# Supplementary material for: Transcriptomic landscape of blood platelets in healthy donors
Source: Sci Rep. 2021 Aug 3;11:15679. doi: 10.1038/s41598-021-94003-z (PMC8333095; doi:10.1038/s41598-021-94003-z)
Supplement: Supplementary file 1 — Supplementary Figures. [file 41598_2021_94003_MOESM1_ESM.docx]

**Scientific Reports | Supplemental Material**

**Transcriptomic landscape of blood platelets in healthy donors**

*Anna Supernat^1^, Marta Popęda^1^, Krzysztof Pastuszak^1,2^, Myron G. Best^3,4^, Peter Grešner^1^, Sjors In 't Veld ^3,4^, Bartłomiej Siek^5^, Natalia Bednarz-Knoll^1^, Matthew T. Rondina^6-9^,* *Tomasz Stokowy^10^, Thomas Wurdinger^3,4^, Jacek Jassem^11^, Anna J. Żaczek^1^*

^1^Laboratory of Translational Oncology, Intercollegiate Faculty of Biotechnology, University of Gdańsk and Medical University of Gdańsk, Gdańsk, Poland; ^2^Department of Algorithms and Systems Modelling, Faculty of Electronics, Telecommunications and Informatics, Gdańsk University of Technology, Gdańsk, Poland; ^3^Department of Neurosurgery, Brain Tumor Center Amsterdam, Cancer Center Amsterdam, Amsterdam UMC, VU University Medical Center, Amsterdam, The Netherlands; ^4^Brain Tumor Center Amsterdam, Amsterdam UMC, VU University Medical Center, Amsterdam, the Netherlands; ^5^Department of History and Philosophy of Medical Sciences, Medical University of Gdańsk, Gdańsk, Poland; ^6^University of Utah Molecular Medicine Program, Salt Lake City, UT; ^7^Department of Internal Medicine, Division of General Internal Medicine, University of Utah, Salt Lake City, UT; ^8^George E. Wahlen Veterans Affairs Medical Center Department of Internal Medicine and the Geriatric Research Education and Clinical Center (GRECC), Salt Lake City, UT; ^9^Department of Pathology, University of Utah, Salt Lake City, UT; ^10^Department of Clinical Science, University of Bergen, Bergen, Norway; ^11^Department of Oncology and Radiotherapy, Medical University of Gdańsk, Gdańsk, Poland

*To whom correspondence should be addressed: dr Anna Supernat, Laboratory of Translational Oncology, Intercollegiate Faculty of Biotechnology, University of Gdańsk and Medical University of Gdańsk, Dębinki 1, 80-211 Gdańsk, Poland, tel. +48 58 349 14 38 anna.supernat@gumed.edu.pl.

**Supplemental Material | Tables**

**Table S1** The detailed list of cases included in the study.

**Table S2** Expression ranking list of all spliced RNA viariants found in platelets. Canonical platelet splice variants, defined according to the gene expression markers for platelets listed by PanglaoDB, are in bold.

**Table S3** Results of expression comparison for the 82 transcripts showing moderate difference between males and females.

**Table S4** Detailed summary of assessment of the variability in platelet transcriptome associated with selected factors. In the first row, the table presents the global similarity among RNA transcript vectors in all subjects enrolled in the study. In the following rows, presented are (1) the values of Kolmogorov-Smirnov statistic (D) based on minimum spanning tree and the associated p-value (pKS) for group-wise comparisons of mean RNA transcript vectors; (2) the values of Wald-Wolfowitz statistic (W) based on the minimum spanning tree and the associated p-value (pWW) for group-wise comparisons of within-group distribution of RNA transcript vectors, and (3) medians and interquartile ranges (IQR) of multidimensional within-group similarity among RNA transcript vectors in compared groups with associated p-value for respective group-wise difference between medians, calculated using permutation testing with 10,000 permutations. Statistically significant differences are indicated in bold, with superscripts explaining the respective threshold level for p-value according to applied Bonferroni correction.

**Supplemental Material | Figures**

**A**


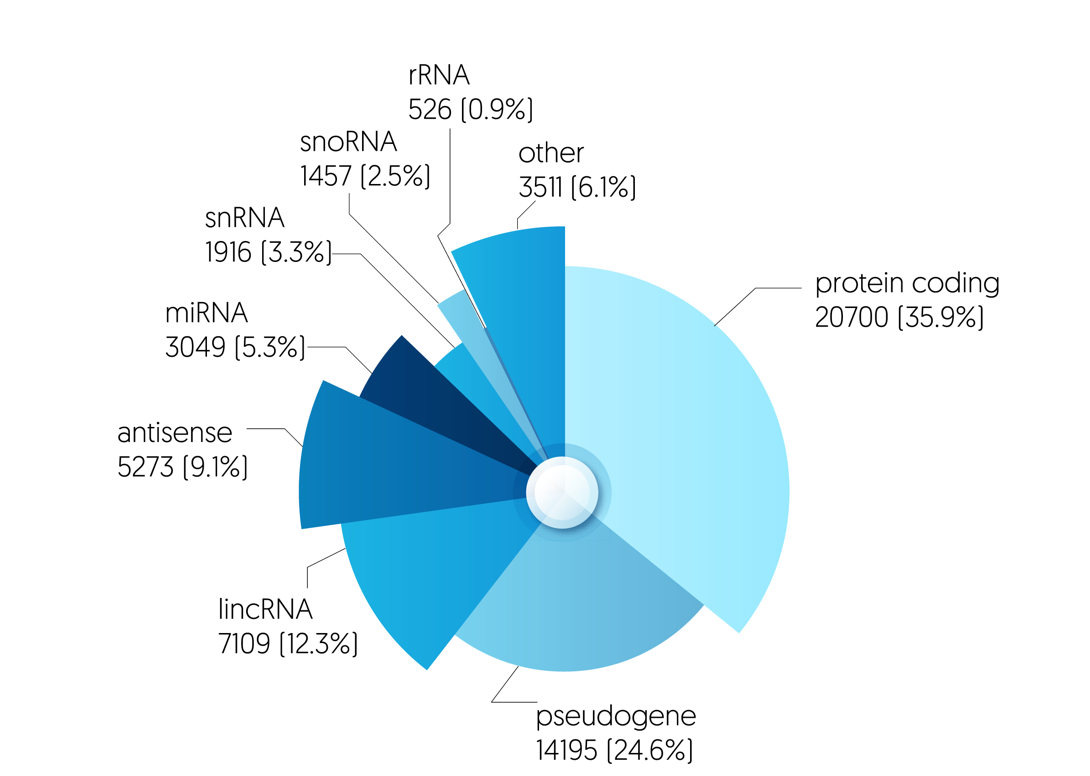


**B**

**
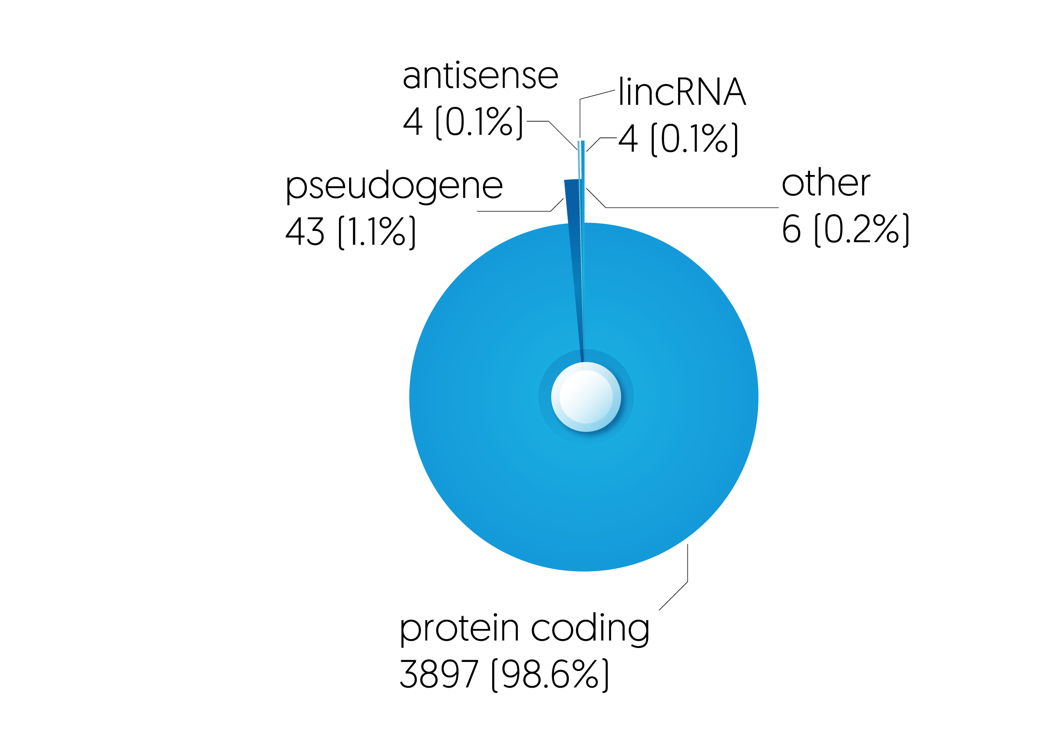
**

**Figure S1** Summary of RNA types found in platelets. **(A)** RNA types before quality control and filtering of low expression reads. **(B)** RNA types after expression profile-based quality control and filtering of low expression reads.


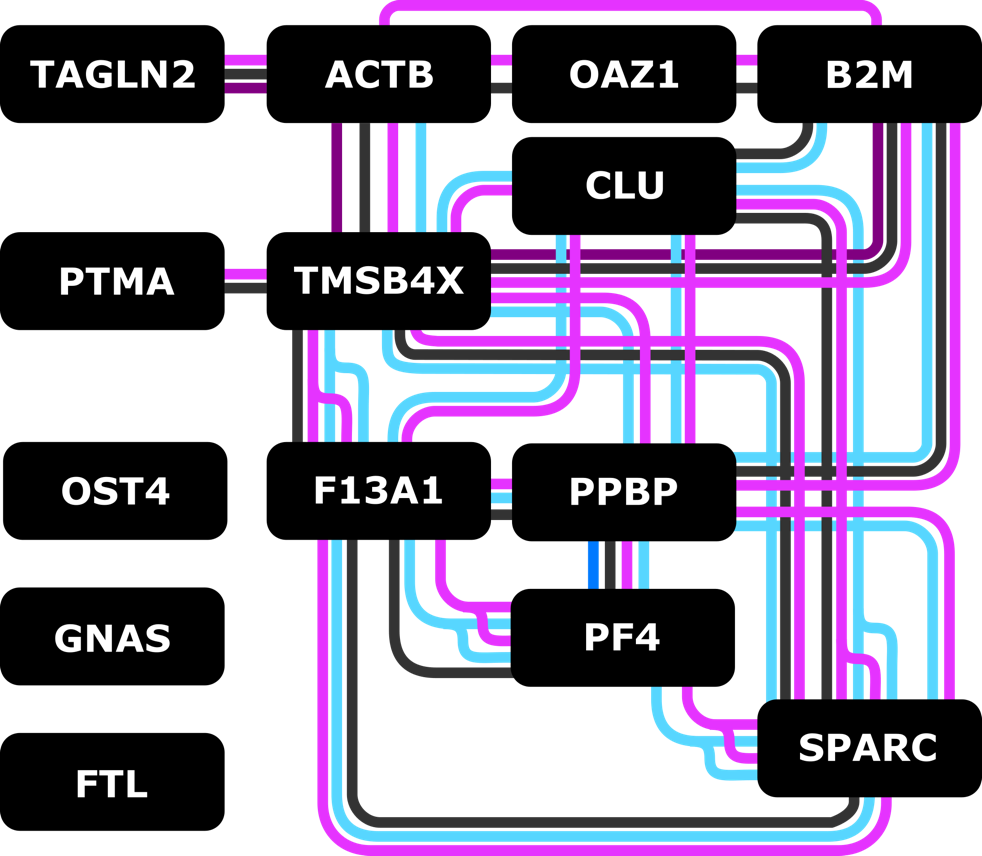


**Figure S2** STRING analysis of the top expressed spliced reads detected in platelets collected from healthy donors. Strong network interaction occurs between the protein products of top expressed transcripts.

**A**


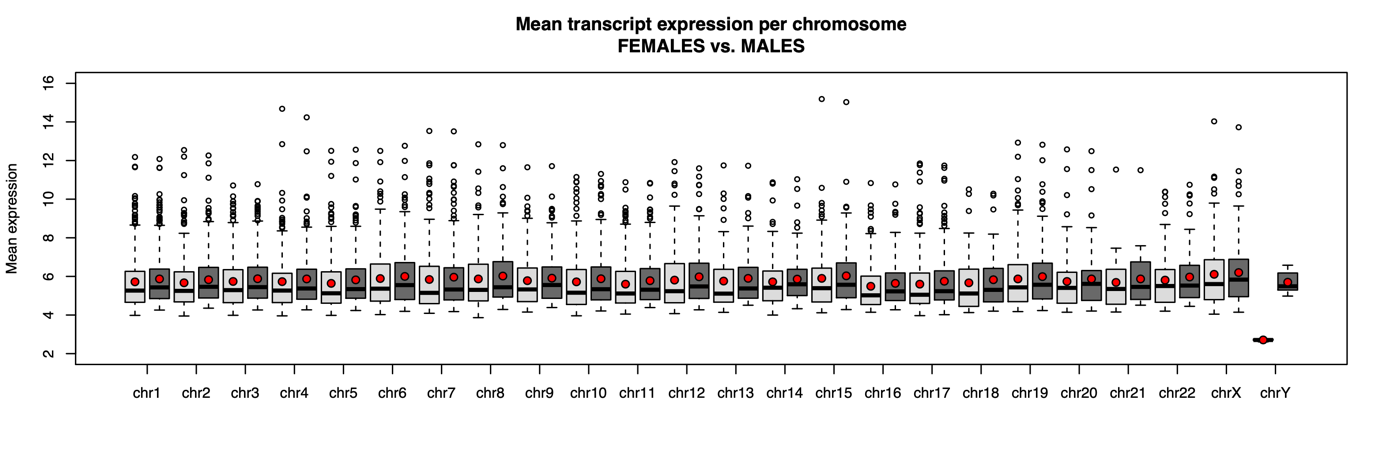


**B**


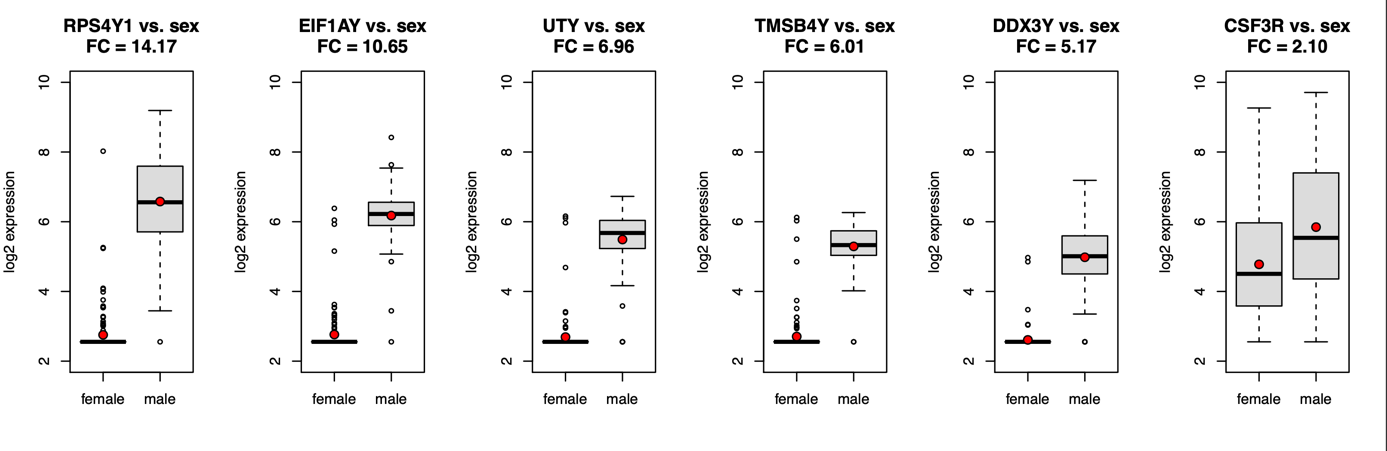


**Figure S3** Splice variant expression dependent on sex. **(A) W**hen analysing the mean transcript expression per chromosome, the only discrepancy between female and male donors is observed for chromosome Y. Points represent mean transcript expression for each chromosome. Light grey boxes depict females, while dark grey boxes represent male donors. **(B)** Charts of chromosome Y splice variants differing according to sex. The major significant differences in platelet splice variants between healthy females and males in the studied cohort were observed for 5 transcripts located on chromosome Y: RPS4Y1, EIF1AY, UTY, TMSB4Y, DDX3Y. In some female donors we observed transcript levels comparable to males. That was most likely caused by mapping misalignment that occurred due to similarity of sequences and alignment of short reads. Such artifacts formation could even intensify in samples with low number of total reads, that undergo normalization process. Additionally, the expression of CSF3R – significantly upregulated in males when compared to females – is shown.


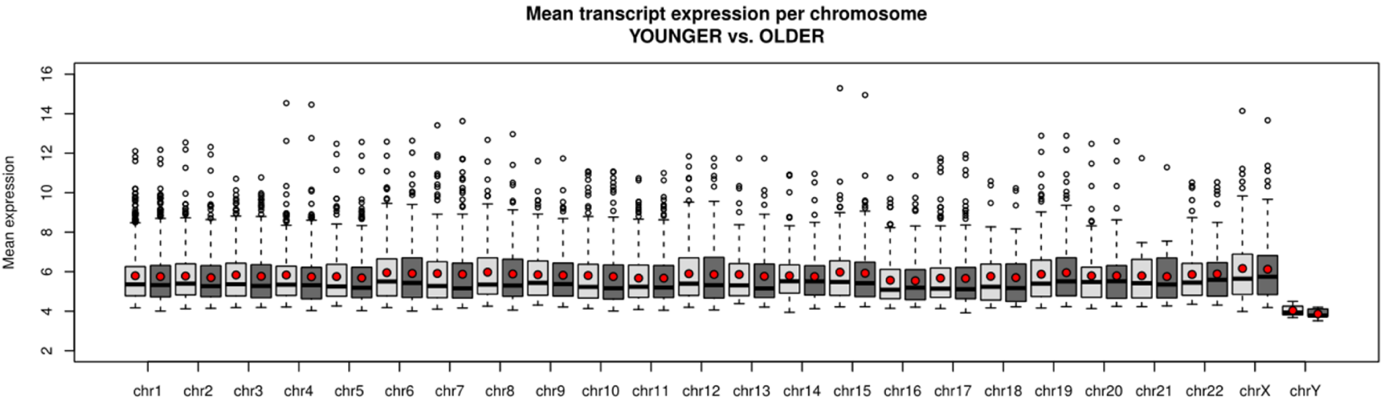


**Figure S4** Splice variant read counts dependent on age. Mean transcript expression per chromosome. No discrepancies can be observed between younger (below 42 years old) and older (above 42 years old) donors. Points represent mean transcript expression for each chromosome. Light grey boxes depict younger, while dark grey boxes represent older donors.


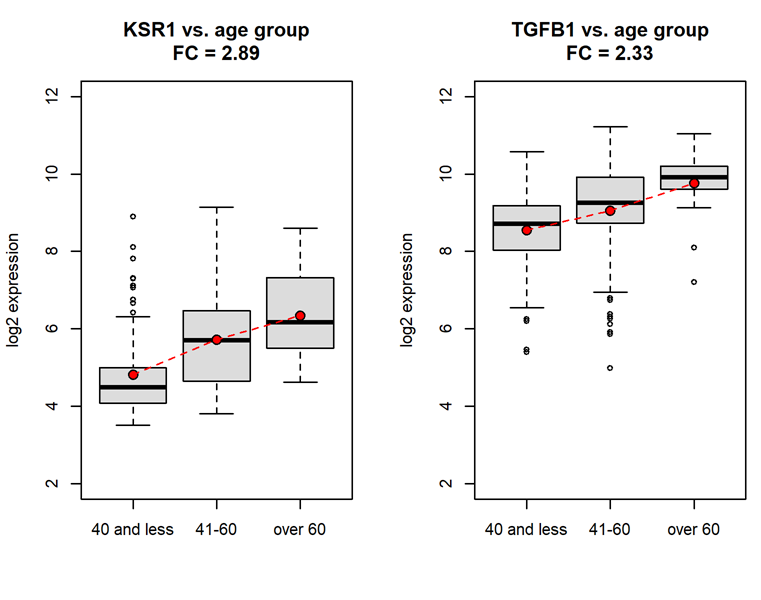


**Figure S5** As age dichotomization yielded few considerable differences, we next performed secondary analyses, dividing the cohort into the following age subgroups: 40 and less, 41-60, and over 60. Charts illustrate the increasing expression levels of *KSR1* (FDR corrected q value < 0.05) and *TGFB1* (FDR corrected q value < 0.05) splice variants. Points represent mean expression in each group.


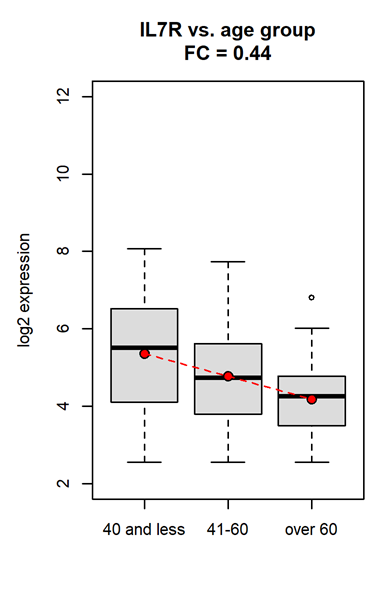


**Figure S6** Chart illustrating the decreasing expression level of *IL7R* gene (FDR corrected q value < 0.05) according to three age subgroups. A moderate decrease with age was observed for 7 genes (0.52-0.67); all FDR corrected q value < 0.05. Points represent mean expression in each group.


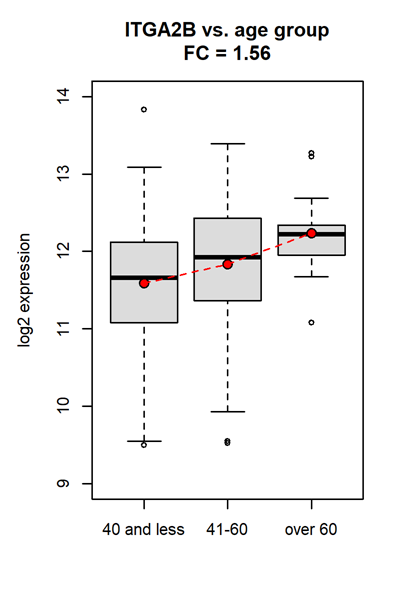


**Figure S7** Chart illustrating the level of platelet-specific ITGA2B splice variant according to three age subgroups (FDR corrected q value < 0.05). Moderate increase of expression is observed. Points represent mean expression in each group. Moreover, 32 further splice variants demonstrated modest increase of expression (FC range 1.50-1.87), while a moderate decrease with age was observed for 7 genes (0.52-0.67); all FDR corrected q value < 0.05. Interestingly, over-representation analysis of the 33 genes with slightly increasing expression level revealed enrichment in focal adhesion.


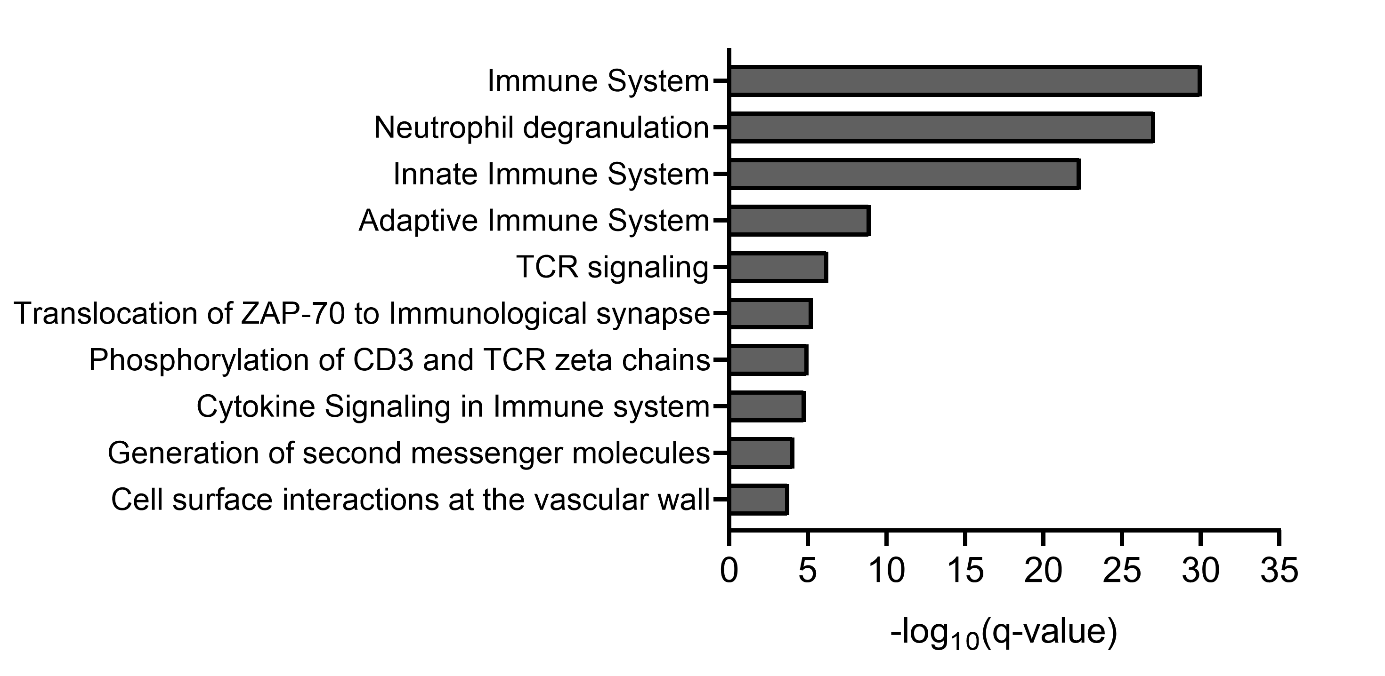


**Figure S8** Functional annotation pathway analysis of differentially expressed genes, with at least 4-fold expression change, upregulated in cluster 2.
